# Editorial: Biomedical application of DNA modifications

**DOI:** 10.3389/fgene.2023.1286185

**Published:** 2023-09-08

**Authors:** Fengbiao Mao, Husile Baiyin, Jinchen Li, Xiao Chen, Yungang Xu, Chenqi Wang, Chang Li

**Affiliations:** ^1^ Institute of Medical Innovation and Research, Peking University Third Hospital, Beijing, China; ^2^ Cancer Center, Peking University Third Hospital, Beijing, China; ^3^ Bioinformatics Center, National Clinical Research Center for Geriatric Disorders, Xiangya Hospital, Central South University, Changsha, China; ^4^ Laboratory of Marine Protozoan Biodiversity and Evolution, Marine College, Shandong University, Weihai, China; ^5^ Department of Cell Biology and Genetics, School of Basic Medical Sciences, Xi’an Jiaotong University Health Science Center, Xi’an, China; ^6^ Center for Global Health and Infectious Diseases Research, College of Public Health, University of South Florida, Tampa, FL, United States

**Keywords:** biomedical application, DNA modifications, human diseases and disorders, DNA methylation, RNA methylation

With the advances of high-throughput sequencing technologies, various DNA modifications have been discovered, such as 5-methylcytosine, 5-carboxycytosine, 5-hydroxymethylcytosine, N6-methyladenine and N7-methylguanine (Raiber et al., 2017). DNA modifications are widespread in different species and are indispensable in multiple biological processes, such as gene regulation and tumorigenesis, genomic imprinting, X-chromosome inactivation and genome stability (Michalak et al., 2019). Furthermore, the decline of sequencing cost have turned DNA modifications to biomedical and clinical applications, which is more consistent with the purpose of precision medicine (Shen et al., 2018; Li et al., 2022). Therefore, making full use of advanced tools to conduct sensitive detection and accurate quantification of these DNA modifications can help us facilitate their biological applications in human diseases.

DNA modification is strongly associated with tumorigenesis and development (Koch et al., 2018). The purpose of this Research Topic is to explore and focus applied research on DNA modification in clinical samples to identify new biomarkers and investigate the underlying mechanisms involved in complex diseases such as human cancers and developmental disorders. Herein, we have collected four relevant articles in our Research Topic based on base modification and genetic mutations. These studies have developed novel computational methods (Ma et al.; Chen et al.) and found cell-free biomarkers (Chen et al.) or genetic variants (Alaamery et al.; Fu et al.) to elucidate the pathology and development of diseases.

Detecting the methylation of cell-free DNA (cfDNA) is a promising approach for early diagnosis and prognosis assessment of human cancers since DNA methylation usually alters in the very early stage of malignant tumors (Luo et al., 2021). Chen et al. intended to find consistent methylation biomarkers that stably existed in both blood and tumors for the early liquid biopsy diagnosis of lung adenocarcinoma (LUAD). Hence, the authors identified 725 differentially methylated CpGs by comparing the genome-wide cfDNA methylation profiles between LUAD patients and healthy donors. Subsequently, they discovered seven CpGs were highly correlated with LUAD risk. Based on the 7-CpGs methylation panel and overall survival information, the authors designed a reliable model to predict tumor prognosis.

Similar to DNA modification, RNA also has a variety of chemical modification types, which play significant roles in biological processes (Li et al., 2016). Previous studies have shown that RNA methylation abnormalities play a key role in the initiation and progression of cancer (Xue et al., 2022). Ma et al. proposed RNA methylation will affect and change the immune infiltrating cells in the tumor microenvironment (TMB), which helps guide the treatment strategy of immune checkpoint blockade (ICB). Based on the expression of 24 potential m^7^G regulators in 817 lung adenocarcinoma (LUAD) patients, authors identified three different m^7^G modification patterns corresponding to immunoapoptosis, immunoinflammation, and immunorejection phenotypes, respectively. Additionally, they established the m^7^Gscore model and evaluated the m^7^G modification pattern of individual LUAD patients. Their results showed that lower m^7^Gscores in patients were associated with poorer prognosis, increased TMB and deficient immune cell infiltration and increased therapeutic response, and vice verse. Thus, the assessment of individual m^7^Gscore could contribute to predicting the clinical response to ICB therapy and adjuvant chemotherapy.

DNA methylation changes are known to lead to an increase in mutation rate, especially for C>T transition and genetic mutations are the determinants underlying human inherited diseases and cancers (Dobre et al., 2021; Wang et al., 2021). For instance, chronic kidney disease (CKD) is a long-term disease condition that causes a great economic burden to society, but the specific pathogenic mechanism remains unclear. Alaamery et al. explored the pathogenesis of CKD by using targeted next-generation sequencing (NGS) to sequence 102 previously reported genes that are associated with CKD, and aimed to clarify the genetic mechanism of end-stage renal disease (ESRD). The NGS panel revealed 13 novel gene variants were significantly associated with ESRD, including four previously unreported genetic variants in any other population.

In addition, balanced chromosome abnormalities (BCAs) are the most common types of chromosomal abnormality and associated with congenital abnormalities in newborns (Talkowski et al., 2012). Fu et al. analyzed the 21 cases with balanced chromosome abnormalities by whole-genome sequencing (WGS) to explore the translocation events and tried to develop a potential prenatal diagnosis method. They found that WGS had higher resolution in breakpoint mapping and high sensitivity in detecting BCA than canonical assays used in clinical practice. Among the 21 fetuses, WGS assay revealed known causative genes were interrupted in two cases. In general, the method proposed by the authors is worthy of reference in prenatal diagnosis and genetic counseling based on BCAs.

In conclusion, the above studies indicated that with the decline of the cost of high-throughput sequencing and increasing computer resources, promising approaches have been established focused on DNA/RNA modification or genetic variants and they will be widely applied to the studies of various diseases. Additionally, with the in-depth understanding of base modification, researchers have designed many editing tools to change the modification status of a given target gene, which is opening up the potential of employing CRISPR epigenome-editing and base-editing technologies to treat and prevent human diseases (Nakamura et al., 2021; Fan et al., 2022).
